# Exploring Neurobehaviour in Zebrafish Embryos as a Screening Model for Addictiveness of Substances

**DOI:** 10.3390/toxics9100250

**Published:** 2021-10-07

**Authors:** Anne Havermans, Edwin P. Zwart, Hans W. J. M. Cremers, Maarten D. M. van Schijndel, Romy S. Constant, Maja Mešković, Laura X. Worutowicz, Jeroen L. A. Pennings, Reinskje Talhout, Leo T. M. van der Ven, Harm J. Heusinkveld

**Affiliations:** Centre for Health Protection, National Institute for Public Health and the Environment (RIVM), P.O. Box 1, 3720 BA Bilthoven, The Netherlands; edwin.zwart@rivm.nl (E.P.Z.); hans.cremers@rivm.nl (H.W.J.M.C.); maarten.van.schijndel95@gmail.com (M.D.M.v.S.); romysconstant@gmail.com (R.S.C.); m.meskovic19@gmail.com (M.M.); laura.xenia@hotmail.nl (L.X.W.); jeroen.pennings@rivm.nl (J.L.A.P.); reinskje.talhout@rivm.nl (R.T.); leo.van.der.ven@rivm.nl (L.T.M.v.d.V.); harm.heusinkveld@rivm.nl (H.J.H.)

**Keywords:** zebrafish embryo, locomotion behavior, nicotine, nicotinic acetylcholine receptor, neuroadaptation

## Abstract

Tobacco use is the leading cause of preventable death worldwide and is highly addictive. Nicotine is the main addictive compound in tobacco, but less is known about other components and additives that may contribute to tobacco addiction. The zebrafish embryo (ZFE) has been shown to be a good model to study the toxic effects of chemicals on the neurological system and thus may be a promising model to study behavioral markers of nicotine effects, which may be predictive for addictiveness. We aimed to develop a testing protocol to study nicotine tolerance in ZFE using a locomotion test with light-dark transitions as behavioral trigger. Behavioral experiments were conducted using three exposure paradigms: (1) Acute exposure to determine nicotine’s effect and potency. (2) Pre-treatment with nicotine dose range followed by a single dose of nicotine, to determine which pre-treatment dose is sufficient to affect the potency of acute nicotine. (3) Pre-treatment with a single dose combined with acute exposure to a dose range to confirm the hypothesized decreased potency of the acute nicotine exposure. These exposure paradigms showed that (1) acute nicotine exposure decreased ZFE activity in response to dark conditions in a dose-dependent fashion; (2) pre-treatment with increasing concentrations dose-dependently reversed the effect of acute nicotine exposure; and (3) a fixed pre-treatment dose of nicotine induced a decreased potency of the acute nicotine exposure. This effect supported the induction of tolerance to nicotine by the pre-treatment, likely through neuroadaptation. The interpretation of these effects, particularly in view of prediction of dependence and addictiveness, and suitability of the ZFE model to test for such effects of other compounds than nicotine, are discussed.

## 1. Introduction

Tobacco use causes many adverse health outcomes such as cancer, cardiovascular and respiratory diseases and is the leading cause of preventable death worldwide [1,2]. Despite widespread tobacco control measures, tobacco use remains substantial at 17.5% of all people above 15 years old, worldwide [2]. Many smokers want to quit, but are unsuccessful; long-term abstinence rates without treatment are estimated at 3–5% [3]. Moreover, tobacco smoking is so addictive that 9.1% of teenagers met diagnostic criteria for nicotine dependence after smoking less than a single pack and nearly 60% became dependent after smoking five packs of cigarettes [4]. Even though nicotine is the main addictive compound in tobacco, other components and additives may also contribute to tobacco addiction [5,6]. For example, sugars can be naturally present and/or added to tobacco and are likely to contribute to dependence potential by contributing to the formation of aldehydes upon combustion [7,8]. Further evaluation of tobacco and smoke components is essential for understanding their impact on tobacco addiction.

Knowledge about the addictiveness of natural tobacco compounds and additives is essential for implementing legislation and for preparing legislators for developments from the tobacco industry. For example, the European Tobacco Products Directive (TPD) prohibits the use of additives that significantly increase the addictiveness of tobacco products [9]. Currently, there is not sufficient evidence to ban specific substances for this reason, and further research in this context is therefore urgently required. Furthermore, the United States Food and Drug Administration (FDA) has recently proposed a regulation to limit the amount of nicotine in cigarettes to a minimally addictive level [10]. This raises the question of whether additives can be used to increase the addictiveness potential of cigarettes, instead of, or in addition to small amounts of nicotine and possibly other addictive tobacco compounds.

Evaluating the addictive potential of substances requires analysis of the integrated function of several components of the nervous system underlying behavior and thus an intact organism. Historically, addictive substances have been studied using animal models, such as self-administration paradigms in rodents [11,12,13]. However, the use of animals in further studies for tobacco research is unwanted in view of, among others, ethical considerations [14]. Nevertheless, there are currently no traditional in vitro models available that sufficiently reflect the in vivo situation to investigate the addictive potential of tobacco additives due to the complexity of the involved nervous system [10,15]. This makes analysis of tobacco components and additives that may contribute to tobacco cigarette dependence challenging and alternative strategies highly desirable.

Currently, the use of the zebrafish (*Danio rerio*) as a research model to study neuroactive drugs is emerging, as it has multiple advantages over traditionally applied animal models [16,17,18,19]. Importantly, the zebrafish embryo (ZFE) until 120 h post-fertilization (hpf) is not protected under animal experimentation laws [20], making their use in line with the 3R principle (Replacement, Reduction, and Refinement of animal experiments) [21]. In addition, many cellular and molecular pathways, including the neurotransmitter pathways, are well conserved between zebrafish and humans [22,23]. Moreover, complex endpoints such as behavior (an important aspect of addiction) can be studied in ZFE [15]. This makes them an attractive research model for the assessment of the addictive potential of tobacco components and additives.

Substance dependence is caused by neuroadaptation, which is a complex adaptation mechanism as a consequence of excessive use of a neuroactive substance like nicotine. For example, nicotine dependence starts with nicotine binding to nicotinic acetylcholine receptors (nAChRs) in the brain. The stimulation of central nAChRs results in the release of a variety of neurotransmitters, depending on receptor subtype and location. Most notably, it is known to induce dopamine (DA) release in the mesolimbic pathway of the brain, which is responsible for reward [24]. Chronic or repeated exposure to nicotine leads to nAChR desensitization, i.e., a state in which the receptors maintain a high affinity to nicotine (or other agonists) but are unresponsive to its binding [24,25]. As a consequence, higher nicotine concentrations are required to achieve the same level of DA release than during initial exposure. In humans and animals, this neuroadaptation manifests as habituation or tolerance; a significantly diminished effect after repeated exposure to the same dose of a substance [24].

We aimed to evaluate behavioral tolerance in ZFE as proxy for the addictive potency of substances. This approach may have the advantage of a high throughput, enabling the testing of many potentially addictive substances, and combinations thereof. Such a model requires an experimental paradigm that involves ZFE behavior that is reliable, quantifiable, reproducible, and executable in a multi-well microtiter plate suitable for the rapid screening of chemical compounds. Recent studies have shown that the ZFE shows a distinct and reproducible pattern of locomotion behavior during light and dark conditions. That is, their activity increases after transitions from light to dark, and decreases after transitions from dark to light [19,26,27]. The mechanism behind this behavior is still unknown, however it has been suggested that the increase in activity after a sudden onset of darkness facilitates navigation back to illuminated areas that allow for finding food [26]. Exposure to neuroactive drugs such as ethanol and cocaine has been shown to alter the locomotor response of ZFE during the light/dark transition test [18,19]. Previous studies have also demonstrated the development of tolerance in ZFE to ethanol and oxazepam [16,28], indicating that the ZFE provides a useful model to study behavioral tolerance as a marker of dependence.

In the current study, we used the behavioral light-dark locomotion test with three exposure paradigms: (1) Acute exposure to nicotine, to determine its potency and effect on ZFE locomotion. (2) Pre-treatment with a nicotine dose range followed by a single dose of nicotine, to determine which pre-treatment dose is sufficient to affect the potency of acute nicotine. It was expected that nicotine-induced neuroadaptation would lead to a reduced effect of acute exposure, i.e., habituation/tolerance. (3) Pre-treatment with a fixed dose combined with acute exposure to a dose range to determine a potential decrease in the potency of acute nicotine exposure after pre-treatment (Table 1). The locomotor experiments were preceded by experiments to determine the kinetics of absorption and clearance of nicotine in ZFE, and explorative studies to optimize the behavioral testing procedure.

## 2. Materials and Methods

### 2.1. Chemicals

All compounds for this study were obtained from Sigma-Aldrich (Zwijndrecht, The Netherlands). (-)-Nicotine (CAS No. 54-11-5) was directly diluted in Dutch standard water (DSW) containing NaHCO_3_ (100 mg/L), KHCO_3_ (20 mg/L), CaCl_2_.2H_2_O (200 mg/L) and MgSO_4_.7H_2_O (180 mg/L)) dissolved in demineralized water.

### 2.2. Housing and Maintenance

Wild-type zebrafish (*Danio rerio*; AB-strain) were obtained from the Karlsruhe Institute of Technology (KIT, European Zebrafish Resource Center, Institute of Toxicology and Genetics, Eggenstein-Leopoldshafen, Germany), and propagated in the RIVM lab. Fish were kept in 8 L Tecniplast ZebTec tanks (Buguggiate, Italy) in a flow-through system. Ambient conditions were a light/dark cycle of 14/10 h, water temperature 27.5 °C ± 0.5 °C, conductivity 500 μS ± 100 μS and pH 7.5 ± 0.5. The fish were fed twice a day with Special Diet Services Small Granules (Tecnilab-BMI, Someren, the Netherlands) and once a day with fresh *Artemia salinas*. Breeding males and females were kept separately for a few days and fed extra *Artemia*. The morning after joining four males and four females in a 1.7 L breeding tank with a sloped interior (Tecniplast, West Chester, PA, USA), reproduction was triggered by the onset of light, and eggs were harvested, rinsed, and checked for fertilization and batch quality (symmetrical development; ≤10% coagulated eggs) under a stereomicroscope. Healthy batches of eggs at 4–32 cell stage were pooled and then divided over Petri dishes with DSW, and kept in an incubator at 28 °C ± 1 °C.

### 2.3. Zebrafish Embryo Toxicity Test

A Zebrafish Embryo Toxicity test (ZFET) [29] was conducted with nicotine to establish its embryotoxic potency (Table 1). In short, scoring lethal endpoints in the original OECD 236 protocol was supplemented with detailed scoring of (1) malformations (teratology), i.e., observable abnormalities in the anatomy, and (2) developmental delays, i.e., the absence of specific developmental hallmarks at a developmental stage where these should be observable. The ZFET was performed in 24-well plates in a static exposure system, starting with embryos at 2–4 hpf (4–32 cell stage), one embryo per well, 10 embryos per test concentration, and four control embryos per plate. Dilution series of the compound were made in embryo medium (see above), and when necessary neutralized to a pH between 6.5 and 8. At 72 hpf embryos were morphologically scored on developmental and teratological endpoints [29].

### 2.4. Chemical Analysis

Absorption and clearance kinetics in ZFE can vary highly between substances (e.g., [30]). To determine these parameters for nicotine, internal concentrations were measured in ZFE at a range of time intervals for up to 24 h after the start of exposure to 30 µM nicotine. Clearance was measured upon transfer of the ZFE to clean medium after 24 h exposure to 30 µM nicotine, at intervals during 8 h. Two samples were prepared for each time point, and each sample consisted of ten pooled ZFEs. At the target time point, samples were sieved, placed in ice-cold DSW, and washed for 15 s. Embryos were then collected in a 1.5 mL Eppendorf tube and all liquid was removed. The tubes were weighed before and after ZFE collection to determine embryo mass per sample. Next, 500 μL of an ice-cold 1:1 mixture of acetonitrile and methanol (ACN:MeOH) was added, and samples were stored at −20 °C before further processing. Following the collection of all samples, the tubes were vortexed for at least 20 s followed by an ultrasonic bath for 20 min, and centrifuged at 14,000× *g* for 10 min at 6 °C. Finally, 150 μL supernatant was transferred to 1.5 mL autosampler vials and stored at −20 °C until quantification. Samples were analyzed using a Shimadzu NexeraX2 liquid chromatography-mass spectrometry (LC-MS). Separation was done using an Acquity UPLC HSS C18 1.8 μM 2.1 × 150 mm column at 30 °C with a flow of 0.4 mL/min. Eluents used were 10 mM ammonium formate and 1:1 ACN:MeOH. Samples were injected (10 μL) in 5% ACN:MeOH, which then rose to and was maintained for two minutes at 100%, before decreasing to 5% again. Consecutively, nicotine was quantified with an ABSciex Qtrap 6500 MS-Triplequad using multiple reaction monitoring in positive mode. Precursor (Q1) and product (Q3) masses of 162.913 and 131.900 Da, respectively, were used for ion selection and quantification.

### 2.5. Locomotion Behavior in the Zebrabox

To assess changes in neurobehavior as a result of chemical exposure, a light-dark transition test was performed using a ZebraBox (Viewpoint, Lyon, France). The testing procedure followed a previously documented protocol [27]. In short, embryos were exposed in a 6-well plate (20 eggs per concentration and solvent control) containing 5 mL of test medium and kept in an incubator at 27.5 ± 0.5 °C up to 120 hpf. Three exposure protocols were applied, i.e., acute exposure (118–120 hpf) without or with pre-treatment (96–104 hpf). In case of pre-treatment, either a pre-treatment concentration range was combined with a fixed acute concentration to optimize the pre-treatment concentration, or a fixed pre-treatment concentration was combined with an acute exposure concentration range, to assess whether a potency shift occurred when compared to the acute nicotine exposure. Before performing the behavioral experiment, a total of twelve (*n* = 12) embryos per concentration were transferred along with 300 µL of test medium to a 96-well plate (1 embryo per well). Following an acclimatization period of 30 min in the light, free-swimming activity was recorded in the ZebraBox during three repeated triggers of light-dark transitions in 10 min periods. Recorded movies were analyzed using a sensitivity setting of 20, and thresholds of 10 (burst) and 1 (freezing), and locomotor activity was evaluated using the Zebralab Quantization software (Viewpoint, Lyon, France) with “time-in-activity” as output. Due to acceleration and deceleration in the beginning and end of the 10 min recording, data variation improved after removal of the first and last 2-min, leaving the middle 6-min section (Figure S2, Table S2). This 6-min section was therefore further used for evaluation in this study.

### 2.6. Data Analysis and Statistics

The morphology and teratology scores obtained from the ZFET as well as concentration-response data from behavior testing were used to perform a benchmark concentration-response analysis using the PROAST software tool (v70.0; RIVM, Bilthoven, The Netherlands, https://www.rivm.nl/en/proast/; accessed on 17 July 2021 [31]), as a package in R statistical software v3.6.0-4.0.0 (RIVM, Bilthoven, The Netherlands). The concentration-response analysis enables the estimation of a benchmark concentration (BMC or critical effect dose (CED)) at a defined critical effect size (CES). A CED_05_ (CED at CES = 5%) was derived for the ZFET to estimate the highest concentration without signs of embryotoxicity and malformations. Behavior data are expressed as average time in activity per minute per embryo. A CED_50_ for behavior testing was used to compare individual experiments and conditions. The estimated CEDs are reported along with the lower (CEDL) and upper (CEDU) bound of their 90% confidence intervals (Table S2). The quality of datasets (data variation) was assessed through the CEDU/CEDL ratio as a measure for width of the confidence interval, which should be lower than 10. Grub testing was applied to identify obvious outliers, which were mostly non-moving embryos, possibly due to damage of the embryo during transfer to the 96-well plate just before the measurement, or sometimes also identifiable as an error in the processing of the movie.

## 3. Results

### 3.1. Embryotoxicity of Nicotine (ZFET)

To avoid morphological, thus non-neurological, effects confounding the parameter of locomotion in behavior experiments, nicotine was tested for morphological changes and embryotoxicity. No lethality was observed after nicotine exposure, up to the highest nominal medium concentration tested (600 µM). The most common morphological changes observed at concentrations of 60 μM nicotine and above were pericardial edema, malformation of the tail, and scoliosis. The average CED_05_ for morphological effects induced by nicotine at 72 hpf was 85.1 µM (90% confidence interval: 22.6–162 μM; *n* = 2; Figure S1, Table S1). Based on these results we used a maximum nominal concentration of 80 µM for acute exposure to nicotine in the behavior experiments.

### 3.2. Kinetics of Nicotine in Zebrafish Embryos

**Figure 1:**
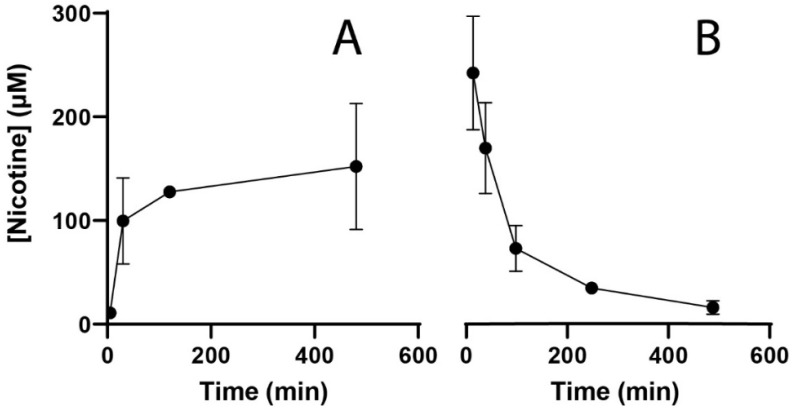
Intraembryonic nicotine concentrations quantified by LC-MS analysis of 10 pooled embryos per sample. (**A**) Measurement of absorption at 5, 30, 120, 480 min after start of exposure to 30 µM nicotine (nominal external concentration). A fast increase in the intraembryonic nicotine concentration is observed, with a plateau phase starting at >2 h. (**B**) Measurement of intraembryonic nicotine concentration at 5, 30, 90, 240, and 480 min after cessation of a 24 h exposure to 30 µM nicotine. Upon cessation of external exposure, the intraembryonic nicotine concentration is cleared fast. Data points represent the average of two independent pooled samples of 10 embryos each.

After the start of exposure, internal concentrations in the embryo increased to levels above the nominal concentration in the medium (30 μM) (Figure 1A), reaching the edge of a plateau (within the analyzed time frame) after about 120 min. When exposure is continued for up to 24 h, the internal nicotine concentration slowly increases further (see starting point Figure 1B). Although a plateau phase is apparent, the apparent continuous increase between 8 and 24 h in combination with internal concentrations above the nominal DSW concentration indicate that bioconcentration occurs. Following the cessation of external nicotine exposure (30 μM nominal concentration) at 24 h, and after changing the medium to clean DSW, nicotine was cleared from the embryos to return to a near background level after 480 min (Figure 1B). These observations indicate that an exposure duration of 120 min (2 h) is sufficient to reach the maximum internal concentration to test for acute effects, while this internal concentration is far higher than the nominal exposure concentration. In addition, a clearance period between pre-treatment and acute exposure should be at least 480 min (8 h) to clear the majority of the intraembryonic nicotine.

### 3.3. Locomotion in the Zebrabox

**Figure 2:**
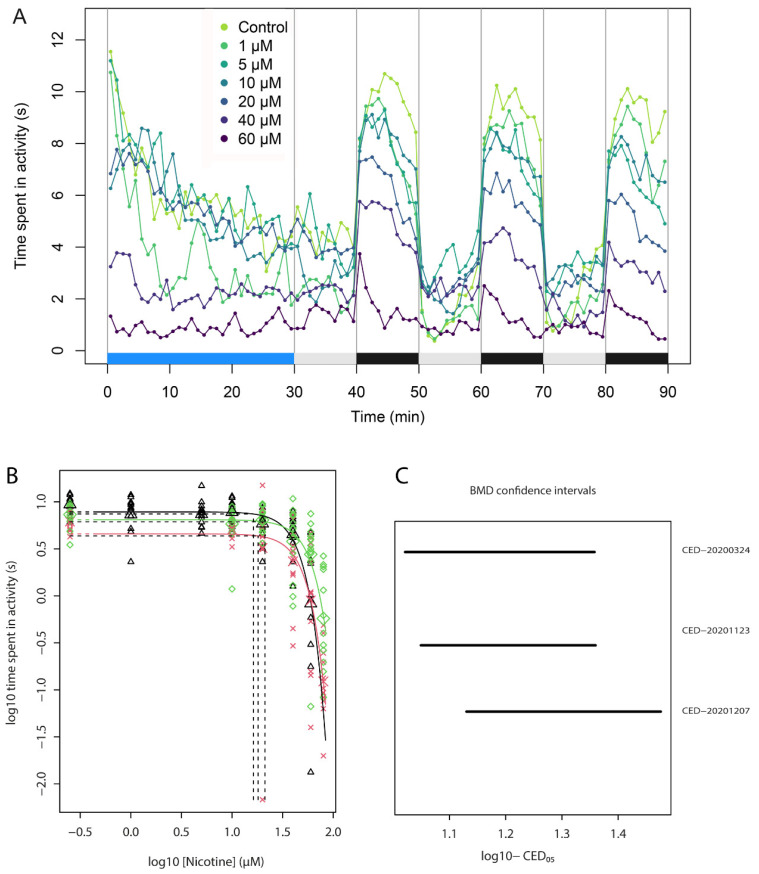
Nicotine exposure concentration-dependently decreases locomotion behavior. (**A**) Example of ZebraBox output visualized in R (v3.6.3), showing time in activity during 30 min acclimatization (blue bar) and three successive 10 min blocks of light (grey) and dark (black). Each dot represents the average cumulative time in activity in the preceding minute of 12 embryos. (**B**) Modelled concentration-response curves for the effect of exposure to nicotine on locomotion. Different colors of curves and symbols represent independent experiments. Symbols are the geometric mean of 12 single embryo data (average time in activity per minute), error bars the 90% confidence intervals. Horizontal dashed lines indicate the 50% effect level (CES) per experiment, vertical dashed lines the resulting CED. (**C**) Confidence intervals (90%) associated with the CEDs from the individual experiments in B.

Exposure of zebrafish embryos resulted in a concentration-dependent decrease in locomotion, as observed from the decrease in time in activity (Figure 2). As can be observed from the example traces in Figure 2A, the pattern of decreasing activity is similar across the three subsequent light-dark blocks. Indeed, analysis of the average data from the second and third blocks indicated no difference in the outcome. Therefore, for further data analysis, we focused on the first light-dark block only. In general, switching off the light immediately increased activity, which gradually decreased again over the 10 min measurement.

### 3.4. Concentration-Response of Acute Nicotine

The effect of acute exposure to nicotine at 120 hpf was tested in three independent experiments in which nicotine reproducibly induced a concentration-dependent inhibition of locomotion (Figure 2B and Figure S3). In these experiments, nicotine showed a similar potency (CED_50_s 41.6–50.1 µM), supported by overlapping confidence intervals (Figure 2C). Based on these experiments, 40 µM was selected as an intermediate dose which induces a distinct effect on locomotion upon acute exposure. This concentration was used in subsequent experiments to assess whether pre-treatment of ZFE with nicotine in a dose range results in a shift in the efficacy of acute exposure.

### 3.5. Effect of Nicotine Pre-Treatment (Concentration Range) on Acute Nicotine Exposure (Fixed Concentration)

**Figure 3:**
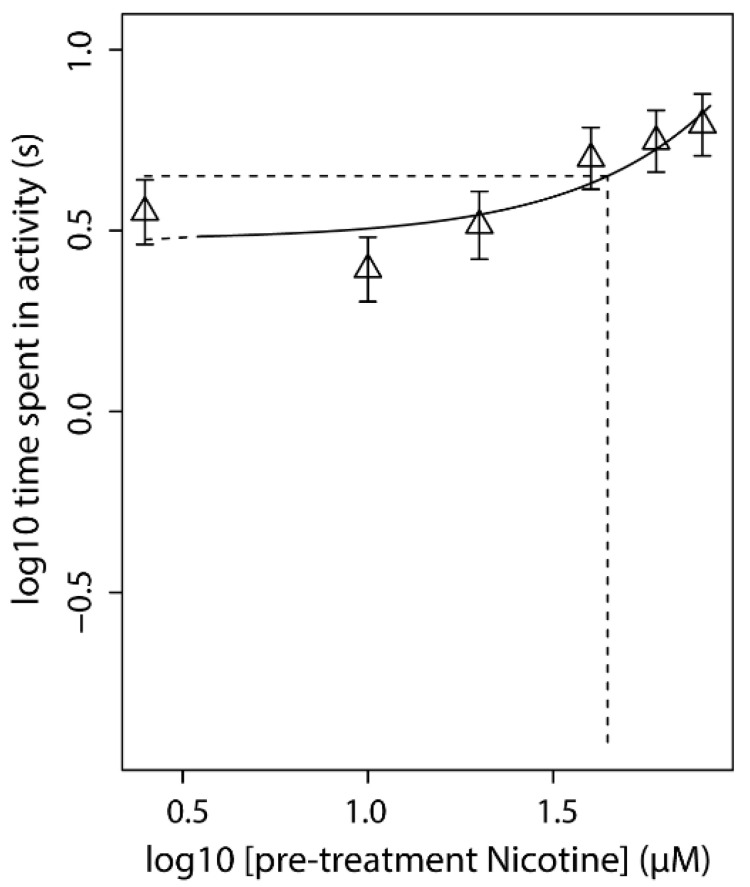
The effect of a concentration-range nicotine pre-treatment (96–104 hpf) on the effectivity of acute exposure to nicotine (40 µM, 118–120 hpf). The graph indicates that with increasing concentration pre-treatment (x-axis), the level of activity increases, demonstrating the decreased potency of acute exposure to nicotine. Symbols represent the geometric mean of *n* = 12 single embryo activity data, error bars indicate the 90% confidence interval. CED (intersection dashed lines) is measured at the 50% effect level (CES). Locomotion was analysed directly after acute exposure.

Pre-treatment of the embryos using a concentration range of nicotine concentration-dependently counteracted the effect of the acute exposure (Figure 3 and Figure S4). This suggests that pre-treatment with nicotine leads to habituation to the compound in a concentration-dependent way. These data indicate that pre-treatment with 30 µM nicotine (near CED_50_) induces a clear change in the response to acute nicotine exposure.

### 3.6. Effect of Nicotine Pre-Treatment (Fixed Dose) on Acute Nicotine Exposure (Dose Range)

**Figure 4:**
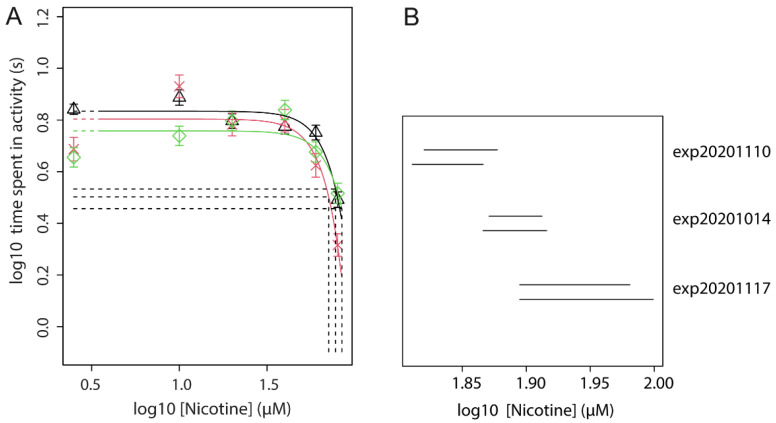
Nicotine pre-treatment reduces the inhibitory effect of acute nicotine exposure on locomotion. (**A**) Combined analysis of acute exposure (118–120 hpf) dose-response data from three independent experiments following pre-treatment (96–104 hpf) to a single concentration. Proast analysis of the data resulted in overlapping confidence intervals (CI) of CED_50_ of the three experiments (**B**), indicating that there are no statistically significant differences between the individual datasets. In B, each pair of CI consists of the CI associated with exponential and Hill models (upper and lower bar, respectively).

**Figure 5:**
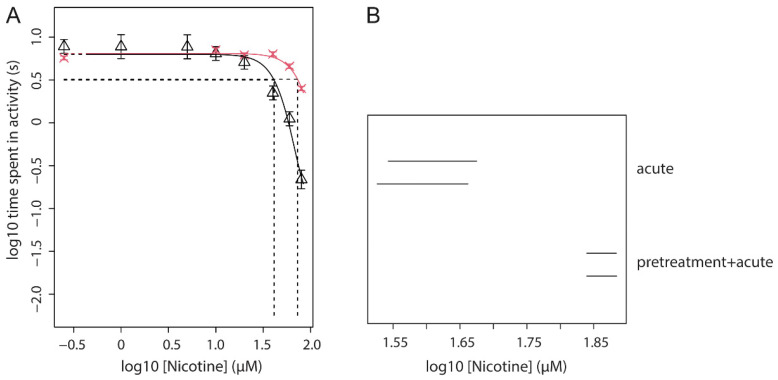
(**A**) Combined analysis of concentration-response data upon acute exposure to nicotine without (black) and with pre-treatment (30 µM; 96–104 hpf; red). Symbols represent the geometric mean of *n* = 12 embryos, error bars are 90% confidence intervals (CI). CED is measured at the 50% effect level (CES; intersection of dashed lines). (**B**) 90% Confidence intervals to the CEDs, where each pair of CI consists of the CI associated with exponential and Hill models (upper and lower bar, respectively).

The combination of a fixed pre-treatment dose of 30 µM nicotine (96–104 hpf) with acute exposure to a concentration-range of nicotine was tested in three independent experiments. Combined concentration-response analysis of these three independent repetitions of the experiment with the experiment as co-variate produced close fits with overlapping confidence intervals for CED_50_, indicating no statistical differences between the experiments (Figure 4 and Figure S5). This analysis showed that the potency of the inhibiting effect of nicotine is decreased when compared to acute exposure to nicotine without pre-treatment, as indicated by the shift in CED_50_ value (CED_50_ Acute only: 41.6–50.1 µM; CED_50_ Acute with pre-treatment: 77.0–84.5 µM). This analysis was repeated in combination with data from the above three experiments of acute nicotine (Figure 2), confirming a lower potency of nicotine when acute exposure is preceded by pre-treatment (Figure 5A and Figure S6). This is indicated by the shift to the right of the pre-treatment + acute fit (red) as compared to acute alone (black), and further supported by a higher CED_50_ (72.9 µM) of the pre-treatment condition as compared to acute alone (CED_50_ = 41.2 µM). In the analysis with exponential models, this difference is statistically significant, given non-overlapping confidence intervals (Figure 5B).

## 4. Discussion

This study aimed to evaluate the use of a ZFE model to study tolerance as a marker of nicotine dependence, potentially to be used as a first-tier screening tool for substance-induced addictiveness. We were able to replicate ZFE locomotion behavior patterns in alternating light and dark conditions in the light-dark locomotion test as found in previous studies [19,26]. In addition, we found that acute exposure to nicotine affected this pattern of behavior, as evidenced by reduced locomotor activity in the dark periods. Although nicotine is a stimulant drug known to increase activity, e.g., [32], depressant effects on locomotor activity have been reported as well in certain experimental conditions; for example, after single dose exposure in rats [33], in adult mice of certain strains [34,35,36,37] and for rats with a high baseline activity [35]. As we applied a single dose of nicotine to animals with high baseline activity, our findings seem in line with previous findings in defined experimental conditions in rodents. Moreover, we demonstrated that repeated exposure to nicotine results in a right-shift of the nicotine dose-response curve, indicative of tolerance. Other studies showing initial depressant effects of nicotine have also demonstrated rapid tolerance development to these effects [33,35]. The mechanism behind these effects has not been conclusively determined, but it has been suggested that decreased activity reflects desensitization of nAChRs, whereas stimulant activity is the result of agonist activation of nAChRs [35]. Moreover, tolerance to the locomotor depressant effects may be mediated by enhanced expression of nAChRs to overcome the diminished cholinergic signaling and restore homeostasis [38,39].

An early stage (alternative) screening tool built to assess potential addictive properties of chemical substances which is able to capture these basic neurobiological process underlying addiction (neuroadaptation), provides a valuable first tier screening. For such a tool to be effective, it requires the presence of a defined array of signaling pathways, including cholinergic and dopaminergic signaling which are generally well-conserved across species [40,41,42,43,44,45]. Fundamental characterization of the (embryonic) zebrafish model has revealed that the cholinergic system is present, with a comparable role and function of the several subtypes of the nAChR to those in humans [17,46]. Subtypes of the nAChR considered to play a role in addictive behavior in humans include the α4β2 and α7 [24,47]. The α4β2 is present in high numbers in the mesolimbic structures of the human brain, mainly the striatum, where it plays a critical role in nicotinic reward and reinforcement through its effect on dopamine release. The α7 is more present in other mesolimbic structures, among which is the hippocampus, and is thought to be involved in somatic withdrawal in humans [48,49]. In contrast to the α7, the α4β2 has a high affinity for nicotine and desensitizes relatively fast [25]. Moreover, the α4 subunits are specifically thought to be involved in tolerance development [50]. Besides their various functions in the brain, nAChRs are present at neuromuscular junctions (NMJ) in the peripheral nervous system, where they facilitate signal transduction of voluntary movement [51,52,53].

While the ZFE possess the same nAChR subtypes that are involved in human addiction (and movement) [46], some specific neuroanatomical structures that are known to be involved in addictive behavior in humans and other animals, (e.g., the nucleus accumbens (NAc), and ventral tegmental area (VTA), have not been described in zebrafish, whereas others have been described (e.g., the habenula) [54]. Despite these differences, adult zebrafish have been shown to display addictive-like behavior in certain test paradigms [16,23]. For example, repeated exposure to nicotine led to a robust conditioned place preference reinforcement response in adult zebrafish, indicative of the establishment of dependence [23]. This effect was related to significant changes in gene expression in pathways and processes also implicated in drug dependence in mammalian models, indicating conservation of some neuro-adaptation pathways between zebrafish and mammals [23]. Moreover, nicotine withdrawal has been described to induce behavioral alterations related to anxiety, motivation and cognition which are similar to those observed in humans and rodent models [55], as well as disruption of shoaling behavior [56]. Other studies using zebrafish models have also confirmed that nicotine affects cognition and anxiety-like behaviors, e.g., [57,58,59,60,61].

In contrast to the conventional in vivo rodent models and adult zebrafish models, certain parts of addictive behavior, such as voluntary drug-taking or seeking, cannot be modelled in zebrafish in the applied setup, where embryonal fish were involuntarily exposed to nicotine in their tank environment. This setup also implies a different route of exposure, i.e., predominantly transdermal, as compared to humans (i.e., inhalation) and other experimental animals (e.g., inhalation, oral ingestion, intravenous, intracerebral).

Nevertheless, the current experimental data indicate the induction of tolerance. However, this strongly suggests neuroadaptation, experiments on receptor expression, and in particular receptor function would be required to confirm this. For example, experiments with selective antagonists such as methyllycaconitine (MLA; α7 nAChR inhibitor) or dihydro-β-erythroidine (DHBE; α4β2 nAChR inhibitor) would provide relevant insights. Pilot experiments at our lab using these inhibitors in the light-dark transition test show that MLA did not cause a dose-response effect, whereas DHBE did, indicating that the α4β2 subtype plays a role in the current readout (locomotion) whereas the α7 subtype does not. This suggests that the behavioral effect of nicotine in our model is a specific receptor-mediated response rather than a consequence of overt toxicity. In addition to the use of receptor agonists, experiments using a fluorescent calcium dye may be applied to visualize the calcium influx as a result of the opening of the nAChRs in the cell membrane upon exposure to nicotine. Desensitization of nAChRs will leave the receptors in a blocked state and this should thus be visible as a decreased influx of calcium. In order to further exclude the possibility that the effects in our study are mediated by peripheral nAChRs, a peripheral nicotine antagonist such as hexamethonium or trimetapham may be applied in future research.

In conclusion, we demonstrate that tolerance as a result of exposure to a known addictive compound, nicotine, occurs in the ZFE. This indicates that one of the basic mechanisms underlying addiction, and ultimately addictive behavior, can be assessed in ZFE. As such, the model can potentially be used to screen for substance-induced tolerance as a proxy for the addictiveness of substances, and mixtures thereof, including combinations of nicotine and other tobacco components and/or additives, such as flavorings. Formal validation of the model would require further testing with comprehensive sets of addictive and non-addictive substances with various underlying mechanisms. For regulatory application, a whole-organism alternative tool such as the ZFE can be of particular value for first-tier screening purposes to decide whether to proceed with e.g., in vivo studies. Alternatively, ZFE could be included in a battery of new approach methodology (NAM) tools, to build and inform toxicological pathways describing exposure to substances leading to addiction in humans. Thus, although further studies are needed to validate the model and confirm the underlying mechanism, the ZFE light-dark transition test has clear potential as a model for screening potentially addictive neuroactive compounds.

## Figures and Tables

**Table 1:** Sequence of toxicological experiments in this study.

	Type of Experiment	Endpoint	Purpose	Result
0	Toxicity testing (ZFET)	embryonal development	Find sub-toxic dose for further testing	Figure S1
1	Dose-range testing of acute nicotine	locomotion	Find potency of acute nicotine and an effective dose for exp. 2	Figure 2
2	Dose-range of nicotine pre-treatment with a fixed effective dose of acute nicotine	locomotion	Find an effective pre-treatment dose for exp. 3	Figure 3
3	Fixed dose of nicotine pre-treatment with a dose-range of acute nicotine	locomotion	Find an effect of pre-treatment on the potency of acute nicotine	Figures 4 and 5

## Data Availability

Data will be made available upon reasonable request.

## Supplementary Materials

The following are available online at https://www.mdpi.com/article/10.3390/toxics9100250/s1, Figure S1: Analysis of embryotoxicity in the ZFET, Table S1: CED’s and confidence bounds at CES = −0.05 (−5%)., Figure S2: Confidence intervals of experiments using the full 10-min Dark block (left) vs. its middle 6-min section, Table S2: BMD confidence bounds at CES = −0.5 (−50%), Figure S3: Concentration-response analysis showing fitted concentration-response curves for the effect of exposure to nicotine on locomotion, Figure S4: The effect of a concentration-range nicotine pre-treatment (96–104 hpf) on the effectivity of acute exposure to nicotine (40 µM, 118–120 hpf), Figure S5: Nicotine pre-treatment reduces the inhibitory effect of acute nicotine exposure on locomotion, Figure S6: Combined analysis of concentration-response data upon acute exposure to nicotine without (black) and with pre-treatment (30 µM; 96–104 hpf; red).
