# Supplementary material for: Exploring Neurobehaviour in Zebrafish Embryos as a Screening Model for Addictiveness of Substances
Source: Toxics. 2021 Oct 7;9(10):250. doi: 10.3390/toxics9100250 (PMC8539716; doi:10.3390/toxics9100250)
Supplement: Supplementary file 1 [file toxics-09-00250-s001.zip › toxics-1388270-supplementary.pdf]

toxics

Supplementary Materials: Exploring Neurobehaviour in Zebrafish Embryos as a Screening Model for Addictiveness of Substances

Anne Havermans, Edwin P. Zwart, Hans W.J.M. Cremers, Maarten D.M. van Schijndel, Romy S. Constant, Maja Mešković, Laura X. Worutowicz, Jeroen L.A. Pennings, Reinskje Talhout, Leo T.M. van der Ven and Harm J. Heusinkveld

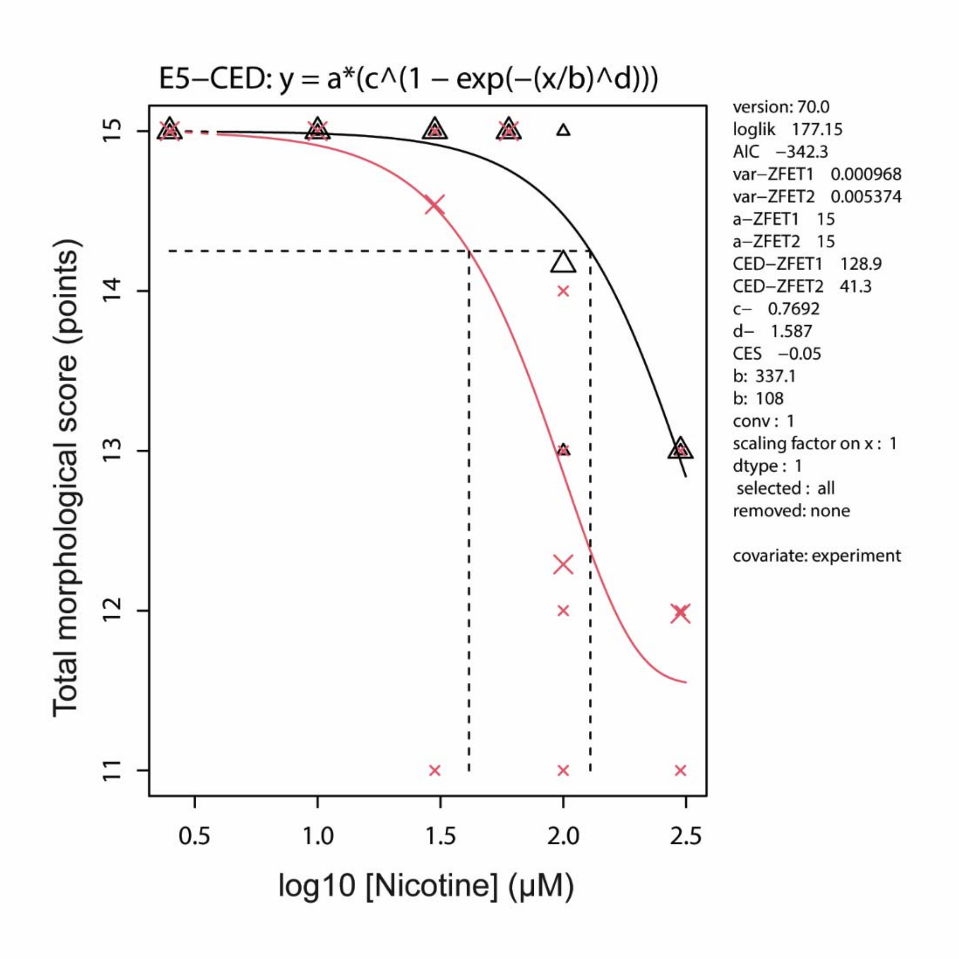

Figure S1. ZFET. A combined analysis of two independent ZFET experiments. Large symbols represent the geometric mean of single embryo data (small symbols). Horizontal dashed line represents the -5% (0.05) effect level (CES), the intersection with the fitted curve determines the CED.

Table S1. CED's and confidence bounds at CES = −0.05 (−5%).

	CED_{05}	lower bound of 90%CI	upper bound of 90%CI
ZFET1	128.9	87.6	162
ZFET2	41.3	22.6	54.7
average	*85.1*		

A comparison was conducted between the full 10-minutes dark-1 block and its middle 6-minutes section of the data underlying Figure 4 (main text), i.e. fixed pre-treatment

followed by acute nicotine in a dose-range. This comparison shows that exp20201014 separates from the other two at the 10-minutes analysis, whereas there is a more gradual overlap at the 6-minutes analysis (Figure S5). Also, the ratio of the highest BMDU and the lowest BMDL of all three experiments is smaller in the 6-minutes analysis as compared to the 10-minutes analysis (Table S1), indicating a lower data variation in the former set. This analysis supports preferred use of the middle 6-minutes section.

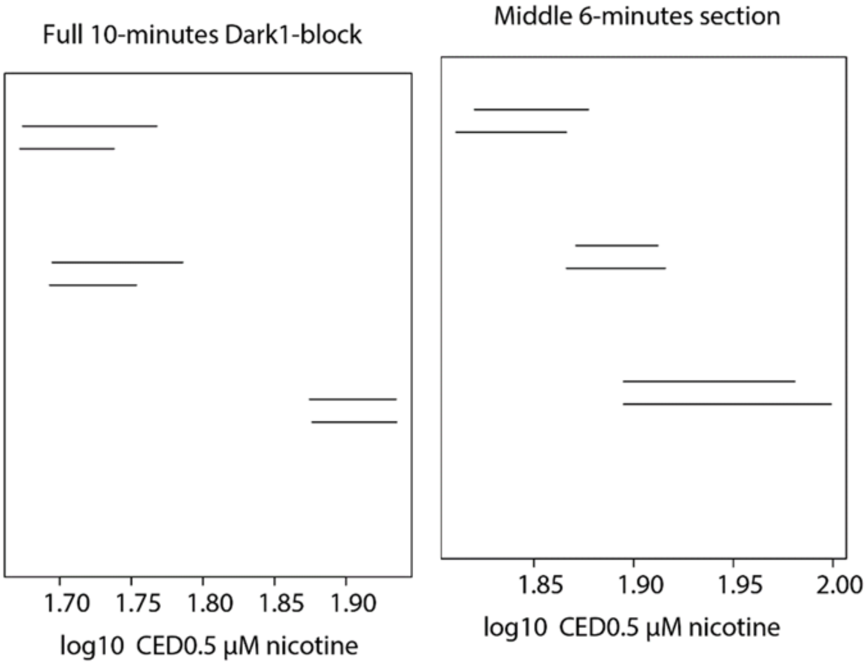

Figure S2. Confidence intervals of three experiments (20201014, 20201110, 20201114, from bottom to top), derived at a 0.5 critical effect size (CES) in a data set of the full first dark 10-minutes block (left) and its middle 6-minutes section. Each pair of bars represents the analysis with exponential (upper) and Hill (lower) models.

Table S2. BMD confidence bounds at CES = −0.5 (−50%).

	**full 10-minutes Dark-1 block**		**middle 6-minutes section**	
**experiment**	**BMDL.lowest**	**BMDU.highest**	**BMDL.lowest**	**BMDU.highest**
1 CED-20201014	74.9	86.2	73.5	82.4
2 CED-20201110	49.3	61.1	64.7	75.4
3 CED-20201117	47	58.6	78.5	99.8
	highest ratio U/L: 86.2/47 = 1.83		highest ratio U/L: 99.8/64.7 = 1.54	

BMDL, BMDU, lower and upper bounds of the 90% confidence interval; BMDL and BMDU were derived from a combined analysis with exponential and Hill models.

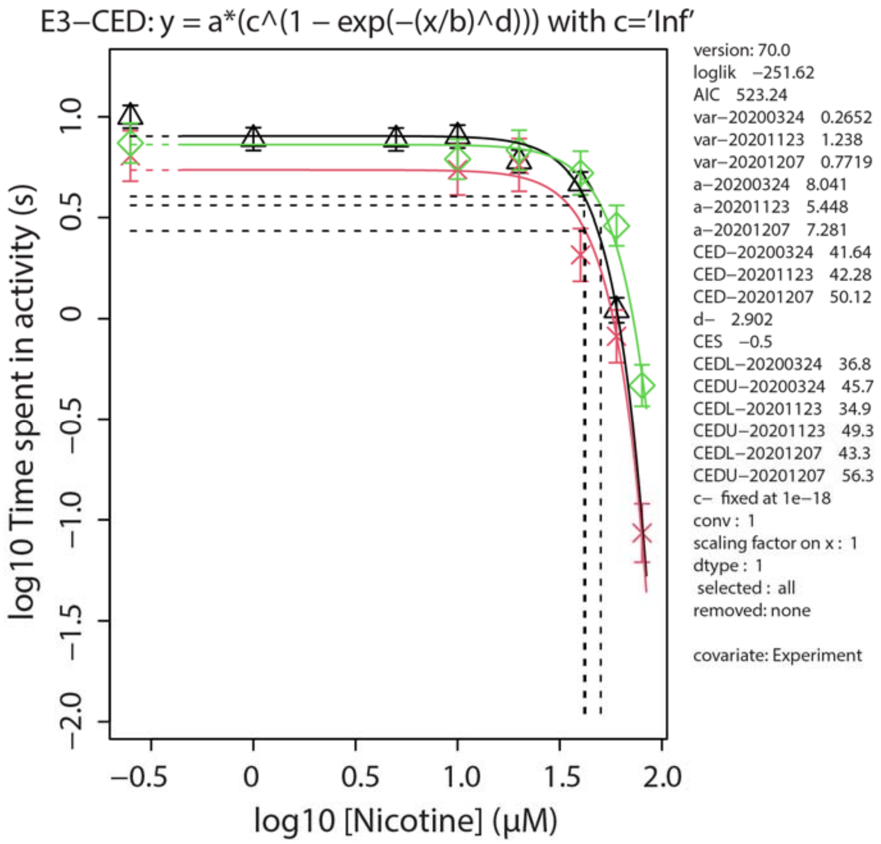

Figure S3. Concentration-response analysis showing fitted concentration-response curves for the effect of exposure to nicotine on locomotion. Different colors of curves and symbols represent independent experiments. Symbols represent the geometric mean of n = 12 single embryo data; error bars are 90% confidence intervals. CEDs were measured at the 5% effect level (CES). Concentration-response data was fitted using the E3-CED model [equation: y = a*(c^(1−exp(−(x/b)^d))) with c = Inf].

Output variables:

Loglik: log likelihood

var: within group variance

a: background response according to the fitted model

d: steepness of the fit

c: maximum response

conv: did the fit algorithm converge? Yes if 1, no if 0.

CED – x: Critical effect dose – experiment identifier

CES: Critical effect size

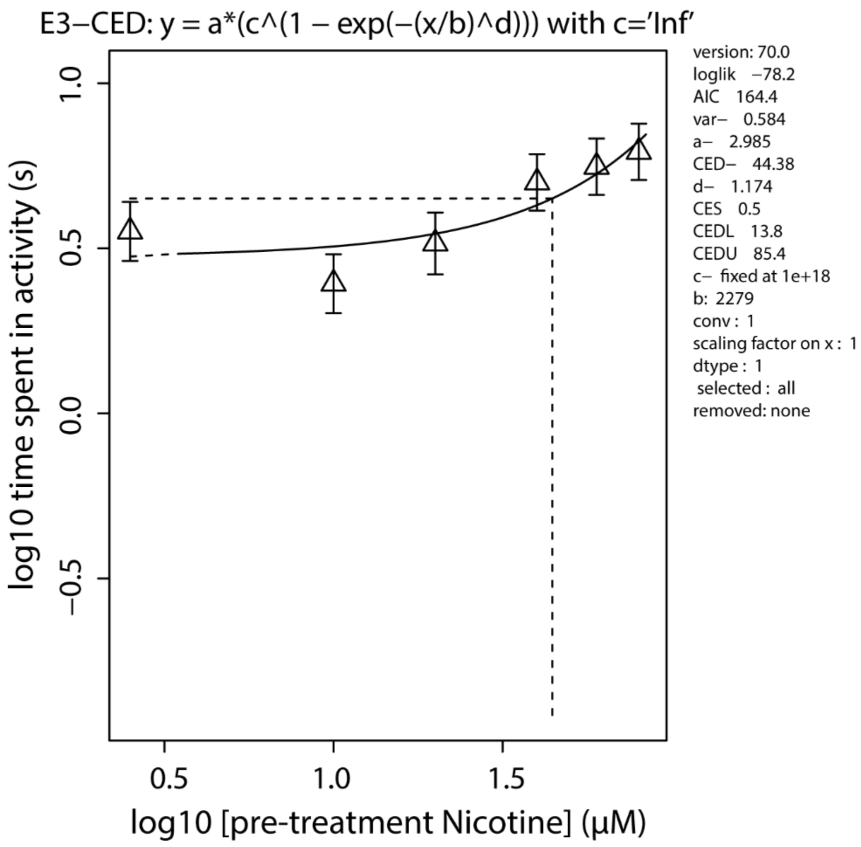

Figure S4. The effect of a concentration-range nicotine pre-treatment on the effectivity of acute exposure to nicotine. The effect of a concentration-range nicotine pre-treatment (96–104hpf) on the effectivity of acute exposure to nicotine (40µM, 118–120hpf). The graph indicates that with increasing concentration pre-treatment (*x*-axis), the level of activity increases demonstrating the decreased potency of acute exposure to nicotine. Symbols represent the geometric mean of $n = 12$ single embryo data; error bars are 90% confidence intervals. CED (intersection dashed lines) is measured at the 5% effect level (CES). Locomotion was analysed directly after acute exposure. Data was fitted using the E5-CED model following the equation: y = a*(c^(1−exp(−(x/b)^d))).

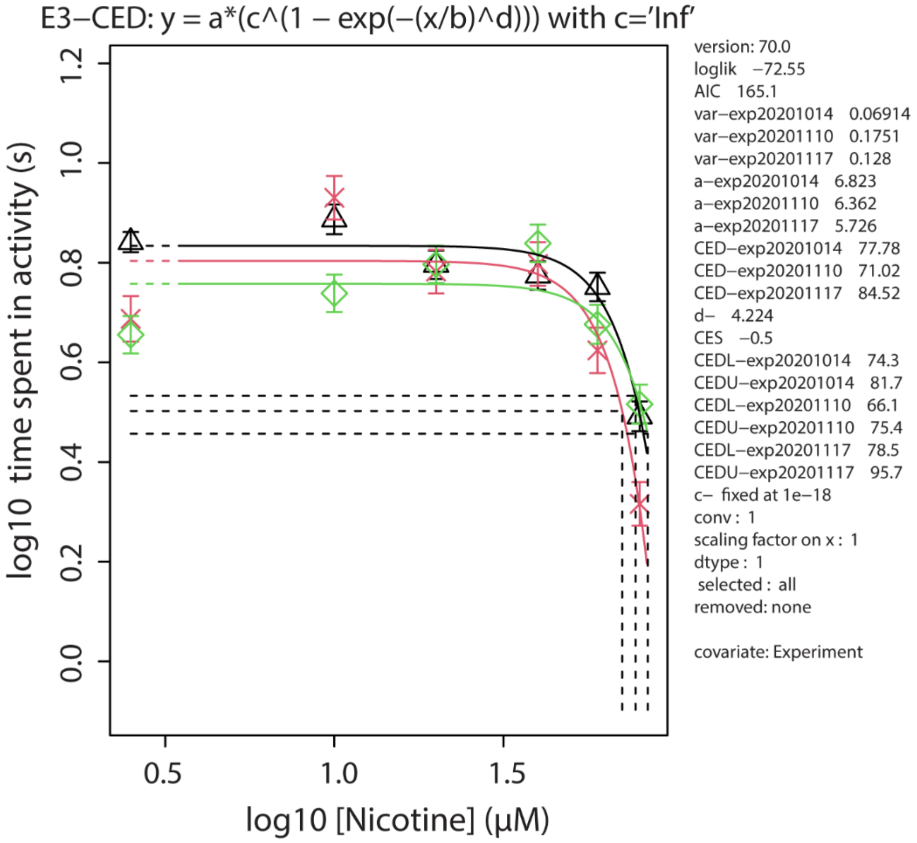

Figure S5. Nicotine pre-treatment reduces the inhibitory effect of acute nicotine exposure on locomotion. Combined analysis of acute exposure (118–120hpf) concentration-response data from three independent experiments following pre-treatment (96–104hpf) to a single concentration. Proast analysis of the data results in a single fit, indicating that there are no statistically significant differences between the individual data sets. Symbols represent the geometric mean of n = 12 single embryo data; error bars are 90% confidence intervals. Data was fitted using the E3-CED model [equation: y = a* (c^(1−exp(−(x/b)^d))) with c = Inf].

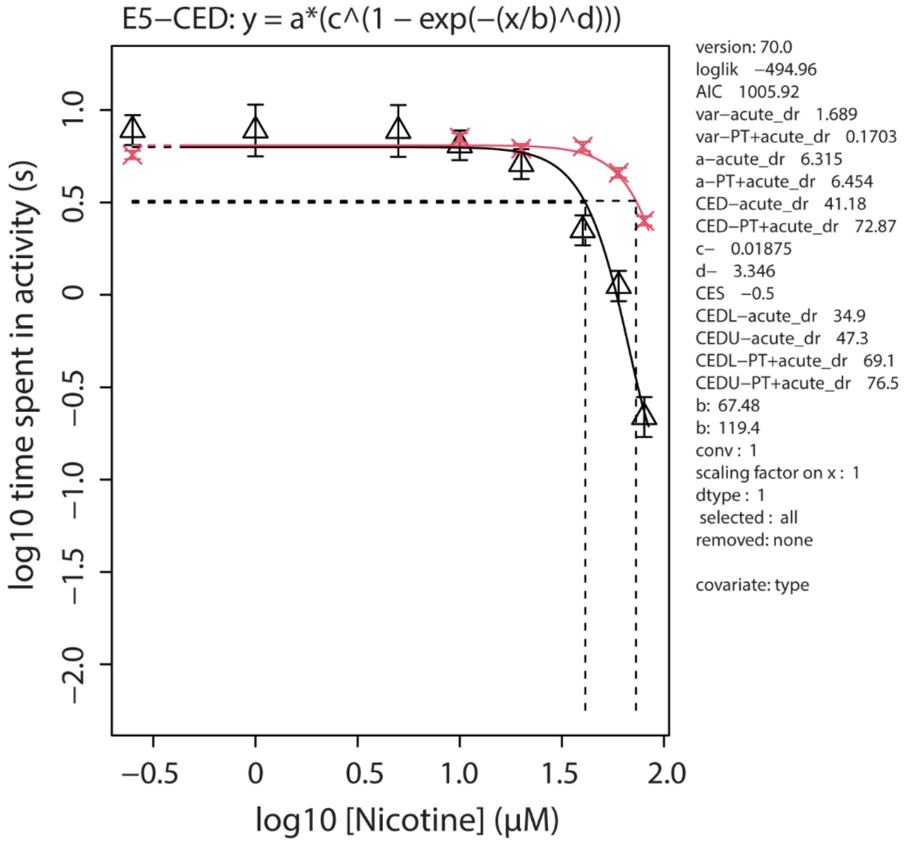

Figure S6. Combined analysis of concentration-response data upon acute exposure to nicotine without and with pre-treatment. Black, acute exposure to nicotine without pre-treatment; red, same with pre-treatment (30µM; 96–104hpf). Symbols represent the geometric mean of *n* = 12 single embryo data; error bars are 90% confidence intervals. CED is measured at the 5% effect level (CES). Data was fitted using the E3-CED model [equation: y = a*(c^(1−exp(−(*x*/b)^d))) with c = Inf].
